# Time-Resolved scRNA-Seq Tracks the Adaptation of a Sensitive MCL Cell Line to Ibrutinib Treatment

**DOI:** 10.3390/ijms22052276

**Published:** 2021-02-25

**Authors:** Viktoria Fuhr, Ehsan Vafadarnejad, Oliver Dietrich, Panagiota Arampatzi, Angela Riedel, Antoine-Emmanuel Saliba, Andreas Rosenwald, Hilka Rauert-Wunderlich

**Affiliations:** 1 Institute of Pathology, University of Würzburg and Comprehensive Cancer Center (CCC) Mainfranken, 97080 Würzburg, Germany; viktoria.fuhr@uni-wuerzburg.de (V.F.); rosenwald@uni-wuerzburg.de (A.R.); 2 Helmholtz Institute for RNA-Based Infection Research (HIRI), Helmholtz-Center for Infection Research (HZI), 97080 Würzburg, Germany; ehsan.vafadarnejad@helmholtz-hiri.de (E.V.); oliver.dietrich@helmholtz-hiri.de (O.D.); emmanuel.saliba@helmholtz-hiri.de (A.-E.S.); 3 Core Unit Systems Medicine, University of Würzburg, 97080 Würzburg, Germany; panagiota.arampatzi@uni-wuerzburg.de; 4 Mildred Scheel Early Career Center (MSNZ), University Hospital of Würzburg, 97080 Würzburg, Germany; angela.riedel@uni-wuerzburg.de

**Keywords:** mantle cell lymphoma, scRNA-seq, ibrutinib, drug resistance

## Abstract

Since the approval of ibrutinib for relapsed/refractory mantle cell lymphoma (MCL), the treatment of this rare mature B-cell neoplasm has taken a great leap forward. Despite promising efficacy of the Bruton tyrosine kinase inhibitor, resistance arises inevitably and the underlying mechanisms remain to be elucidated. Here, we aimed to decipher the response of a sensitive MCL cell line treated with ibrutinib using time-resolved single-cell RNA sequencing. The analysis uncovered five subpopulations and their individual responses to the treatment. The effects on the B cell receptor pathway, cell cycle, surface antigen expression, and metabolism were revealed by the computational analysis and were validated by molecular biological methods. The observed upregulation of B cell receptor signaling, crosstalk with the microenvironment, upregulation of *CD52*, and metabolic reprogramming towards dependence on oxidative phosphorylation favor resistance to ibrutinib treatment. Targeting these cellular responses provide new therapy options in MCL.

## 1. Introduction

Mantle cell lymphoma (MCL) represents a rare subtype among B-cell non-Hodgkin’s lymphoma (NHL) accounting for 3–10% of adult-onset NHL in the western world [1]. Besides the typical translocation t(11;14)(q13;q32) causing Cyclin D1 overexpression, the specific MCL marker SOX11 is overexpressed in most cases [2,3,4]. These features characterize the classical MCL subtype showing localization of aberrant B cells in lymph nodes or at extranodal sites prone to progress into even more aggressive blastoid or pleomorphic variants due to high risk for oncogenic abnormalities [5]. While the “watch and wait” strategy is applied to the indolent forms, symptomatic patients undergo chemotherapy or immunochemotherapy. A high-dose cytarabine-based treatment including rituximab followed by autologous stem cell transplantation serves as an aggressive therapy option for the young cohort, whereas the main subgroup of elderly patients receives a less aggressive drug combination [6]. Even though, the progression-free survival (PFS) can be prolonged by Rituximab maintenance, relapses occur inevitably at all ages [7,8]. Therefore, targeted therapies in MCL are investigated more intensively [9]. Ibrutinib belongs to the class of Bruton tyrosine kinase (BTK) inhibitors representing promising novel agents [6]. By blocking the BTK, B cell receptor signaling and consequently the activation of downstream NF-κB signaling, presumed to function as an essential proliferation stimulation pathway in MCL, are impeded [10,11,12]. Wang et al. reported a satisfactory response rate in pretreated MCL patients [13]. However, even the responding patients will relapse suggesting that some tumor cells evade treatment [14].

Single-cell RNA sequencing (scRNA-seq) has emerged as a powerful tool to profile the transcriptomic landscape of thousands of tumor cells and decipher drug responses with unprecedented resolution [15,16]. The single-cell approach allows the identification of distinct subpopulations bearing differing risk for aggressiveness, metastasis, or drug resistance [17,18]. As these features either already exist prior to therapy or are acquired during increasing chemotherapy pressure, the comparison of tumor cells collected at different therapy phases may uncover the origin of resistance [19]. Wang et al. investigated bone marrow samples from one multidrug resistant MCL patient and reported a decreased immunogenicity and marker genes involved in various drug resistance mechanisms in the malignant B cells [20]. Longitudinal single-cell transcriptomic analysis is currently an interesting topic in the mantle cell lymphoma field to unveil drug resistance mechanisms. However, little is known about the time-resolved response of MCL cells to ibrutinib monotherapy at the single-cell level.

Here, we studied the transcriptomic landscape of a well characterized MCL cell line model REC-1 across ibrutinib treatment [21]. Despite sensitivity to ibrutinib, 20% of the MCL cell line REC-1 can persist a five-day treatment indicating higher resistance of this subpopulation to BTK inhibition. We investigated the surviving cells of untreated (Ctr), 6 h, and 48 h ibrutinib treated samples by scRNA-seq. As pharmacokinetic studies revealed a maximal achievable clinically relevant concentration of 408 nM and with respect to titration studies in REC-1, the cells were treated with 400 nM ibrutinib [21,22,23]. In order to capture the early response to ibrutinib on a transcriptomic level, we chose the 6 h time point. The 48 h time point identified the adaptations of cells surviving the initial drug response. Additional validation assays were performed to corroborate the scRNA-seq data.

## 2. Results

### 2.1. High Reproducibility of Replicates

**Figure 1:**
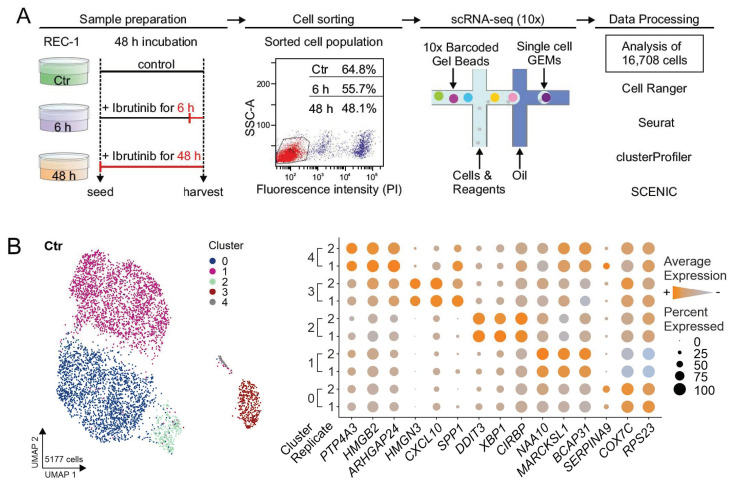
Overview of scRNA-seq approach and evaluation of reproducibility. (**A**) REC-1 cells were cultured for 48 h in total, one sample stayed untreated (Ctr), the second was treated for 6 h and the third for 48 h with ibrutinib (400 nM). Scatter plot (SSC-A vs. fluorescence intensity (propidium iodide)) of 48 h is shown representative for all treatments, cells of gate P1 (red) entered scRNA-seq (relative proportion of included cells are indicated for each sample). Droplet-based single-cell RNA sequencing was performed using the 10x Genomics platform with encapsulation of single cells in GEMs (gel bead-in-emulsions). The data was analyzed implementing Cell Ranger (10x Genomics), and R packages such as Seurat, clusterProfiler, and SCENIC; (**B**) uniform manifold approximation and projection (UMAP) representation visualized the five subpopulations in the aggregated (replicate 1 and 2) and cell cycle regressed data set of Ctr and the corresponding dot plot shows the expression of selected top 10 marker genes for every subpopulation; size of dots refers to the percentage of cells expressing the gene, color intensity represents the average expression level.

The single-cell RNA sequencing experiment was performed across two independent biological replicates including a control of untreated REC-1 cells (Ctr) and REC-1 treated with 400 nM ibrutinib for 6 h and 48 h. Side-by-side viability assays ensured the previously shown cell death of REC-1 cells after ibrutinib treatment (data not shown) [21]. Cell sorting, sequencing, and quality control left a total number of 16708 cells for analysis (Figure 1A, Table S1).

To reveal whether both replicates of Ctr reflected similar cell populations, the data was aggregated, clustered, and visualized by uniform manifold approximation and projection (UMAP) technique (see Materials and Methods—Data Analysis). The Ctr sample comprised 5177 cells expressing an average of 3382 genes (Figure 1B, Table S1). The effect of the cell cycle was mitigated implementing Seurat’s cell cycle regression approach to investigate underlying sources of heterogeneity *(*Figure S1). Overall, we could delineate five transcriptionally distinct clusters (cluster 0–4). These subgroups were consistently present as indicated by the equal expression of their marker genes in both biological replicates, confirming the high reproducibility of the two data sets (Figure 1B).

### 2.2. Heterogeneity of REC-1

**Figure 2:**
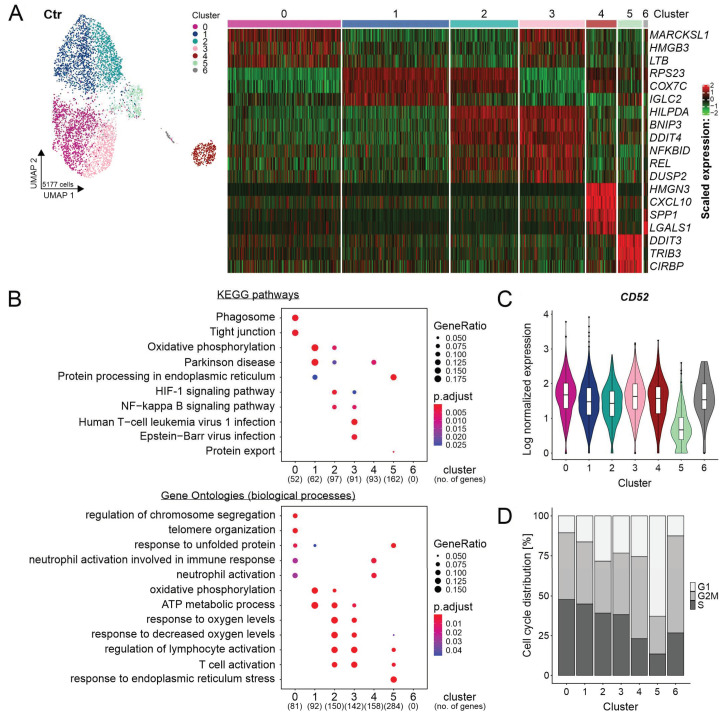
Heterogeneity of REC-1 cell line on the single-cell level. (**A**) Uniform manifold approximation and projection (UMAP) representation visualizing 7 clusters at resolution 0.4 in the integrated and cell cycle regressed data set of untreated cells (Ctr) and the corresponding heatmap showing selected top 10 marker genes (see Table S2 for cluster markers); (**B**) gene set enrichment analysis for Kyoto Encyclopedia of Genes and Genomes (KEGG) pathways and gene ontologies (biological processes) including genes of Ctr with a log fold change > 0.1 and adjusted *p*-value < 0.001 (no. = number); no matches were detected for cluster 6 due to few differentially expressed genes (DEGs); (**C**) violin plot of *CD52* expression in Ctr (clustering is shown in (**A**); and (**D**) bar plot showing the proportions of predicted cell cycle phases (G1, G2/M, or S) across clusters of Ctr by Seurat’s cell cycle scoring.

To study the heterogeneity of REC-1 cells in the Ctr sample, the integrated data was employed. Unsupervised clustering detected seven clusters (cluster 0–6) (Figure 2A).

The clusters 1 and 2 comprised 42% of Ctr cells and showed common marker genes of mitochondrial and ribosomal origin such as *RPS23* and *COX7C* (Figure 2A). Although cluster 4 (7% of Ctr cells) showed increased expression of these markers as well, it was separated owing to the additional expression of genes encoding for the nucleosome binding protein HMGN3, chemokine CXCL10, the extracellular matrix protein SPP1 (Osteopontin), and the proangiogenic galectin-1 encoded by *LGALS1* (Figure 2A, Figure S2A). The clusters 0 and 3 (43% of Ctr cells) were characteristic for the expression of *MARCKSL1*, *HMGB3*, and *LTB* encoding for an actin binding protein, for a chromatin binding protein, and for a cytokine, respectively (Figure 2A). Apart from the gene expression pattern separating the clusters 2 and 3, they shared the markers *HILPDA*, *BNIP3*, *DDIT4*, *NFKBID*, *REL*, and *DUSP2* (Figure 2A). The Kyoto Encyclopedia of Genes and Genomes (KEGG) pathway analysis linked these genes to a higher activity of NF-κB signaling leading to the upregulation of the NF-κB associated genes *NFKBID*, *REL*, and *DUSP2* (Figure 2B). The same cohort of cells showed a metabolism associated with hypoxia (*HILPDA*, *BNIP3*, and *DDIT4*) and dependent on glycolysis (*SLC16A3*) (Figure 2A, Figure S2B). After reclustering of the cluster 4, the differential gene expression according to glycolysis/hypoxia became apparent in this subgroup leading to the separation into two clusters (Figure S2C). On the contrary, cells of cluster 1 were assigned to a metabolism based on oxidative phosphorylation (OXPHOS) (Figure 2B). The upregulation of *DDIT3*, *TRIB3*, and *CIRBP* defined cluster 5 (6% of Ctr cells) (Figure 2A). The interplay of these genes suggested an altered regulation triggered by nutritional, endoplasmic reticulum stress, and unfolded protein response (Figure 2B). Compared to the other clusters, cluster 5 expressed least *CD52*, a favored target for lymphoma therapy (Figure 2C), and contained the highest amount of non-cycling cells assigned to G1 (Figure 2D) [24]. Regarding the low information content of only 1% of Ctr cells, cluster 6 was not included into further functional analyses.

This data showed heterogeneity of REC-1 cells caused by distinct properties of clusters and a further differentiation due to prevalent glycolysis/hypoxia related metabolism.

### 2.3. Evolution and Common Response of Subpopulations

**Figure 3:**
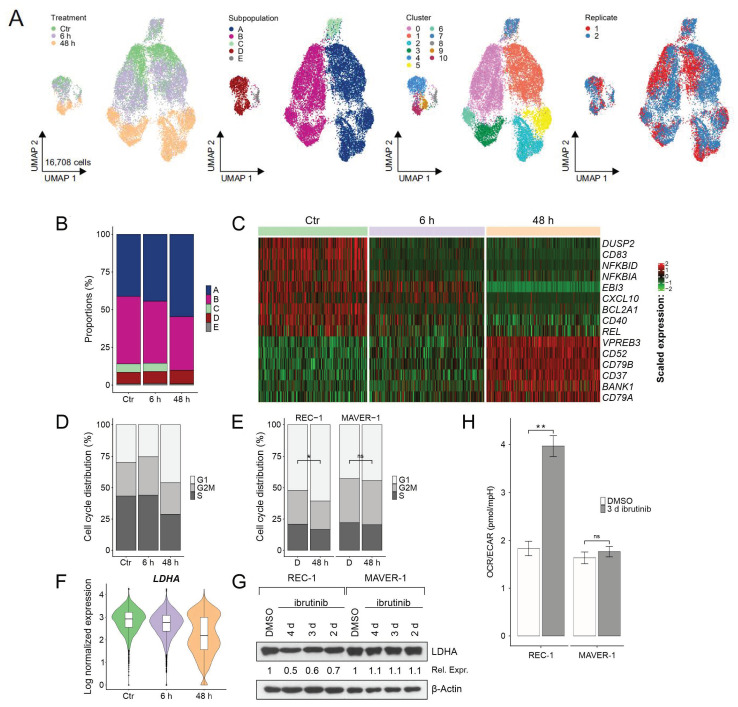
Evolution of subpopulations across ibrutinib treatment. (**A**) Uniform manifold approximation and projection (UMAP) representations of the combined data set including replicate 1 and 2 of Ctr, 6 h, and 48 h after cell cycle regression (clusters are shown for resolution 0.4 with additional subclustering of subpopulation D at resolution 0.3); (**B**) proportions of subpopulations across treatment; (**C**) heatmap of selected marker genes of Ctr, 6 h, and 48 h (see Table S3 for treatment markers); (**D**) distribution of predicted cell cycle phases (G1, G2/M, and S) across treatment by Seurat’s cell cycle scoring; (**E**) distribution of cell cycle phases acquired by flow cytometry (sensitive REC-1 compared to resistant MAVER-1 cell line, D = DMSO control, 48 h = 48 h 400 nM ibrutinib treatment; *n* = 3, * *p* ≤ 0.05, ns = not significant); (**F**) violin plot of *LDHA* expression levels of the single-cell sequencing data; (**G**) Western blot of LDHA expression after ibrutinib treatment (DMSO as control, 400 nM ibrutinib for 2 d, 3 d, and 4 d) in sensitive REC-1 and in resistant MAVER-1, β-actin served as loading control, relative expression (Rel. Expr.) to DMSO control was calculated after normalization to β-actin (Western blot and relative expression values are shown representative for three independent replicates); and (**H**) extracellular flux analysis of 3 d ibrutinib (400 nM) or DMSO (control) treated cells (sensitive REC-1 compared to resistant MAVER-1) by Agilent Seahorse XF 96 Analyzer; the ratio of oxygen consumption rate (OCR) to extracellular acidification rate (ECAR) is shown (*n* = 3, ** *p* ≤ 0.005, ns = not significant).

The combined data set included both replicates of the three treatments (Ctr = untreated, 6 h, and 48 h ibrutinib) (Figure 3A). After cell cycle regression, 16,708 high-quality cells with mean 3035 expressed genes were investigated in downstream analysis (Table S1).

The UMAP plot visualized the transcriptional change of five subpopulations across treatment (Figure 3A). As the gene expression patterns of Ctr still resembled mostly the ones of 6 h for subpopulation A and B, the enclosed cells clustered together. In contrast, the clusters shifted from 6 to 48 h indicating great transcriptomic alterations triggered by longer ibrutinib incubation periods. To investigate the evolution over time in detail, the subpopulations were reclustered revealing the separation of Ctr/6 h from 48 h cells for subpopulation D as well (Figure S3A).

All subpopulations persisted following ibrutinib treatment except subpopulation C disappearing in 48 h. While A and D increased, the proportion of B decreased across treatment (Figure 3B).

Although each subpopulation developed independently, a common response to ibrutinib was determined in all cells of 48 h (Figure 3C). Indeed, NF-κB target genes (*DUSP2*, *CD83*, *BCL2A1*, and *CXCL10*), and *REL* encoding for a NF-κB-subunit, NF-κB regulating genes (*NFKBID* and *NFKBIA*), and *CD40* were downregulated already after 6 h treatment (Figure 3C). In 48 h, the upregulation of B cell receptor associated genes (*CD79A*, *CD79B*, and *VPREB3*) and genes involved in B cell receptor signaling (*BANK1*) followed. Besides the B cell receptor, the expression of the genes encoding for the surface markers CD52 and CD37 increased in all surviving cells after 48 h treatment (Figure 3C). Flow cytometric analysis of REC-1 cells confirmed increased CD52 and CD37, and decreased CD40 protein expression only in sensitive cells after 3 day ibrutinib treatment (Figure S4). In contrast, MAVER-1 representing an ibrutinib-resistant MCL cell line did not alter the expression of these surface antigens.

Comparing the ratio of cells assigned to the G1, G2/M, or S phase revealed an increase of G1 cells in 48 h (Figure 3D). This effect was validated by flow cytometric analysis of fixed and propidium iodide stained cells. The proportion of cells cycling in G1 (with single DNA amount) increased solely in the sensitive REC-1 cells, whereas ibrutinib did not impact the cell cycle of resistant MAVER-1 (Figure 3E).

Focusing on the aforementioned glycolysis and hypoxia associated cells unveiled their continuous development from Ctr towards 6 h and 48 h. While they clustered together with the rather OXPHOS related cells in Ctr/6 h of subpopulation A, B, and D, they diverged into separated clusters in 48 h, in one smaller, glycolysis/hypoxia (*BNIP3* and *DDIT4*) associated and one larger OXPHOS cluster (Figure S3A,B). This effect was caused by the distinct expression of several glycolysis/hypoxia related genes. *LDHA*, *IGFBP2*, and *PGK1* expression switched to two distinct levels across ibrutinib treatment leading to two metabolic species in 48 h, one subgroup with higher and one with lower glycolytic activity (Figure 3F, Figure S3C). The altered expression of *LDHA* was confirmed by Western blot revealing decreased LDHA protein levels after prolonged ibrutinib exposure time in sensitive REC-1 (Figure 3G). Moreover, the metabolic shift was reflected by measuring oxygen consumption rate (OCR) and extracellular acidification rate (ECAR) after 3 d ibrutinib treatment. The ratio of OCR/ECAR raised in the sensitive cell line significantly (Figure 3H) by a decrease of the ECAR (data not shown), suggesting the survival of a population with greater dependence on OXPHOS. On the contrary, ibrutinib resistant MAVER-1 did not alter their metabolic activity (Figure 3H).

### 2.4. Gene Regulatory Networks during Ibrutinib Treatment

Single-cell regulatory network inference and clustering (SCENIC) was applied to investigate the gene regulatory network (GRN) in REC-1 cells and its adjustments triggered by ibrutinib.

**Figure 4:**
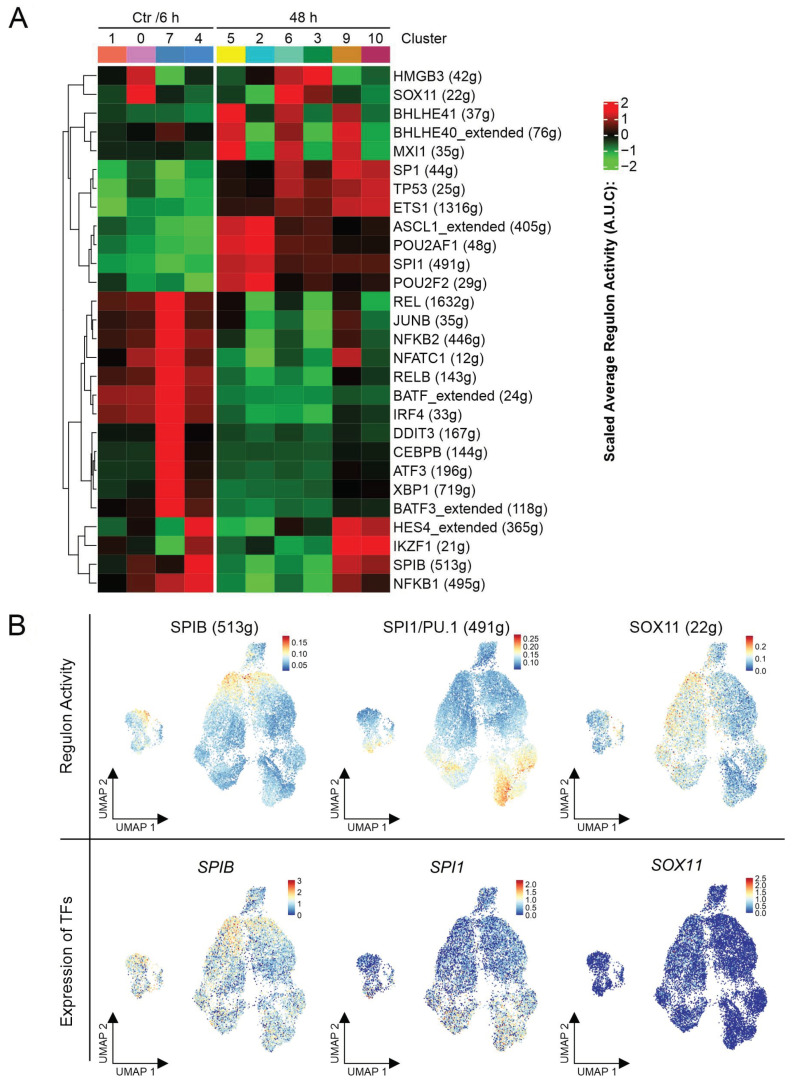
Altered gene regulatory networks during ibrutinib treatment. (**A**) Heatmap representing the alterations in gene regulatory networks in the eleven clusters of the combined analysis (Ctr, 6 h, and 48 h) (clustering is shown in Figure 3A), numbers in brackets indicate the amount of genes forming the gene regulatory networks of the indicated transcription factor and (**B**) uniform manifold approximation and projection (UMAP) representations in the first row show the regulon activity of the indicated transcription factors, UMAPs underneath display the gene expression (log normalized) of the transcription factors.

The heatmap visualized the shift of the GRN across treatment (Figure 4A shows an excerpt, Figure S5 displays the entire heatmap). In accordance with the Seurat analysis, NF-κB transcription factors (NFKB1, REL, NFKB2, and RELB) were more dominant in Ctr/6 h cells. Only the glycolytic/hypoxic cells of subpopulation D (cluster 9) maintained slight activity of NFKB1 in 48 h. The GRNs of TP53, SP1, and ETS1 distinguished the surviving cell population after 48 h ibrutinib treatment. Due to its functions in mediating apoptosis, DNA repair, and cell cycle arrest, TP53 represents a tumor suppressor frequently mutated in MCL. ETS1 activates metastasis or invasion associated genes, while SP1 is involved in essential biological processes like cell proliferation, apoptosis, and differentiation. Furthermore, the influence of several transcription factors (TFs), playing essential roles in proper B cell function, changed across treatment. SPIB, IRF4, and BATF represented specific TFs for the Ctr/6 h cells. After 48 h ibrutinib treatment, SPI1 (*SPI1* gene encodes for PU.1), POU2F2, and POU2AF1 prevailed (Figure 4B).

Subpopulation C comprising only Ctr/6 h cells was marked by the activity of DDIT3, CEBPB, ATF3, and XBP1 referring to the cellular stress response.

A unique GRN was assigned to subpopulation B due to a pattern of HMGB3, a chromatin-binding protein, and SOX11, a neural transcription factor. In general, REC-1 cells were reported to be *SOX11* positive [25]. Referring to our analysis, B exhibited greater activity of the TF SOX11 than the other subpopulations, even though the gene expression of the transcription factor was not enhanced in this subgroup (Figure 4B).

The subpopulation D was characterized by activity of the TF HES4 encoded by a NOTCH1 target gene, and IKZF1, involved in B cell differentiation and function. Furthermore, SPIB activity is solely maintained in this subpopulation in 48 h.

A concordant GRN pattern emerged in the glycolytic/hypoxic part of A, B, and D (cluster 5, 6, and 9). The transcription factors MXI1, BHLHE41, and BHLHE40 share the ability to be upregulated due to hypoxia. This observation supported the uncovered coexistence of two metabolic species (OXPHOS vs. glycolysis/hypoxia) in the Seurat analysis.

SCENIC unveiled a gene regulatory shift in cells from Ctr/6 to 48 h caused by reduced NF-κB activity and changing dominance of important B cell associated transcription factors, and special GRNs characterizing the subpopulations.

## 3. Discussion

Although ibrutinib improved the outcome of relapsed/refractory MCL patients, resistance remains a major challenge in therapy. Reasons for resistance to the BTK inhibitor ibrutinib are diverse and include activation of the alternative NF-κB pathway, missense mutation at the BTK binding site (*BTK*^C481S^), and sustained PI3K/AKT signaling [11,26]. Though the MCL cell line REC-1 is ibrutinib sensitive, a small proportion of cells survive the five-day treatment [21]. These cells may harbor or gain specific features leading to reduced vulnerability to ibrutinib. Therefore, we dissected the transcriptional response of REC-1 cells to ibrutinib over time by scRNA-seq.

Across ibrutinib treatment, all surviving cells underwent similar transcriptomic adaptations. A fast response was the downregulation of NF-κB signaling. In the further course of treatment, the surviving cells increased the expression of B cell receptor subunits, *CD79A* and *CD79B*, representing a potential feedback mechanism to compensate for the weakened BCR signaling and the resulting impeded NF-κB signaling. These modifications were accompanied by a changeover in crucial gene regulatory networks of B cells. As SPIB with its partner IRF4 were reported to promote NF-κB signaling [27], the reduced activity of these TFs following ibrutinib treatment forced the cells to sustain proper BCR signaling by enhancing SPI1, sharing partially redundant functions with SpiB in mouse models [28,29].

Besides *CD79A* and *CD79B*, *CD37* levels were enhanced. Even though the exact functions of this surface glycoprotein are poorly understood, therapeutics were already under investigation to treat NHLs. Pagel et al. included MCL patients to study the effect of Otlertuzumab, a humanized anti-CD37 protein therapeutic, on relapsed/refractory NHLs, resulting in a poor response in this subtype [30]. Apart from upregulated surface antigens, we detected the downregulation of the gene encoding for the tumor necrosis factor (TNF) receptor CD40. Upon CD40 stimulation canonical and noncanonical NF-κB signaling pathways can be activated, whereas *CD40* itself represents a NF-κB target gene [31]. Therefore, we trace the decreased levels to the impaired B cell receptor signaling by BTK inhibition.

Our findings correspond to the previously observed downregulation of NF-κB signaling triggered by ibrutinib in a scRNA-seq approach investigating peripheral blood mononuclear cells from chronic lymphocytic leukemia (CLL) patients collected at different therapy phases. Additionally, the downregulation of *CD52* and *CD37*, and reduced activity of the transcription factor SPI1/PU.1 were determined over a 240-day treatment period by Rendeiro et al., whereas we detected an upregulation of *CD52*, *CD37*, and increased activity of SPI1/PU.1 in our MCL cell line model after 48 h ibrutinib treatment [32].

Single-cell analysis unveiled the heterogeneity that is dominating the transcriptional landscape of REC-1 cells. The heterogeneity of cancer cell lines was already reported with respect to the recurrent existence of cellular programs like cell cycle, stress, and interferon responses amongst others in cell lines originating from diverse tumor entities [33]. Here, a subpopulation of cells showed activity of a HES4 regulated gene network indicating active NOTCH1 signaling [34]. Unique features such as *CXCL10*, *SPP1*, and *LGALS1* suggested a subtype with aggressive features, higher potential of metastasis, promotion of angiogenesis, and immune escape [35,36,37,38,39,40]. These characteristics might determine a specialized MCL population, conserved in the REC-1 cell line, interacting with the microenvironment to build a protective niche. As MCL cells localize not only at nodal sites, but also in extranodal compartments like bone marrow or the gastrointestinal tract, the interplay between tumor cells and microenvironment are implicated and associated with tumor growth and drug resistance leading to re-emergence of the disease after therapy [41].

Another small subpopulation comprising 6% of the untreated REC-1 population characterized by weakest *CD52* expression succumbed solely to 48 h ibrutinib treatment. As all surviving cells upregulated *CD52* over ibrutinib treatment, this surface antigen represents a promising target. Alemtuzumab, an anti-CD52 monoclonal antibody was applied successfully to B cell chronic lymphocytic leukemia (B-CLL) patients since 2001, but was only secondarily tested in MCL without a break-through [42,43]. A study investigating the combination of ibrutinib and alemtuzumab in patients diagnosed with advanced-phase CLL was terminated due to severe adverse events, however, an efficient response was determined [44].

Distinct metabolic states contributed to an additional source of heterogeneity, which strengthened during ibrutinib treatment. While Zhang et al. postulated, that metabolic reprogramming to oxidative phosphorylation empowers MCL cells to evade ibrutinib [45], the theory of the Warburg effect links increased glycolysis to greater tumor growth [46]. Contrary to the reported higher basal OCR/ECAR ratio values of resistant MAVER-1 compared to the sensitive REC-1 [45], the here observed basal OCR/ECAR ratio values did not differ significantly in the two cell lines. Nevertheless, the decreased level of LDHA and the increased OCR/ECAR ratio of the surviving population of sensitive cells after ibrutinib treatment supports the observation of a metabolic shift towards greater dependence on oxidative phosphorylation in cells with limited LDHA activity by Fantin et al. [47]. Our data encourage the approach of targeting the OXPHOS pathway with an ETC complex I inhibitor like IACS-010759 in MCL patients relapsing from ibrutinib. This is currently tested in a phase 1 clinical trial for refractory lymphoma (NCT03291938) and reported to negatively impact proliferation of ibrutinib-resistant MCL cell lines [45].

In this study, we combated confounding or noisy scRNA-seq data, resulting from potential transcriptional burst events induced by drug exposure, dropout events, or other technical variation, performing two independent biological replicates [48]. To prove our findings on the protein level, we studied the effects of ibrutinib by molecular biological methods, considering the delay between altered mRNA and corresponding protein levels [49]. Indeed, we confirmed the greater proportion of quiescent cells after 48 h, and the altered regulation of the surface antigens CD52, CD37, and CD40 and the reduced glycolytic capacity of the surviving cell population with pronounced dependence on oxidative phosphorylation after 3 d ibrutinib treatment. As these observations might arise from indirect effects, we compared the results with the effects on a resistant MCL cell line characterized by less dependence on the impaired classical NF-κB pathway by ibrutinib [50,51]. Thus, we could show that the detected adaptations were unique to the sensitive cell line.

As our work focused on the transcriptional landscape at early stages of treatment, longer incubation periods would reveal whether the observed alterations persist and define a resistant cell population, even though the time frame is restricted due to feasible cell culture conditions. Despite the limitation to a single cell line, the transfer of promising validated in vitro results to the clinics will further push towards new therapeutic opportunities in MCL. Moreover, in future scRNA-seq studies of primary material originating from patients before, during, and after relapse from ibrutinib treatment, the acquired findings from established cell lines might serve as starting points to understand the heterogeneous data sets and might be reflected in a subgroup of patients.

## 4. Materials and Methods

### 4.1. Cell Lines and Reagents

The REC-1 (ACC 584) and MAVER-1 (ACC 717) cell lines were acquired from Deutsche Sammlung von Mikroorganismen und Zellkulturen (DSMZ, Braunschweig, Germany). Cells were cultured in RPMI1640 (Thermo Fisher Scientific, Waltham, MA, USA) supplemented with 10% (REC-1) or 20% (MAVER-1) heat-inactivated fetal bovine serum (Gibco, Thermo Fisher Scientific, Waltham, MA, USA) and 2 mM L-Glutamine (Pan Biotech, Aidenbach, Germany) at 37 °C with 5% CO_2_ in a humidified atmosphere. Cells were regularly tested for mycoplasma (Venor GeM OneStep kit, Minerva Biolabs, Berlin, Germany) and are cultivated in a master/working stock system.

Ibrutinib was purchased from Selleck Chemicals (Absource Diagnostics, Munich, Germany). Primary antibodies LDHA (#3582), β-actin (#4970), and secondary horseradish peroxidase (HRP)-conjugated anti-rabbit IgG antibody (#7074) were obtained from Cell Signaling Technologies (Danvers, MA, USA). CD52-PE (#130-123-972), CD40-PE (#130-111-063), and CD37-PE (#130-123-329), and the human IgG1 isotype control (#130-113-438) were purchased from Miltenyi Biotech (Miltenyi Biotech, Bergisch Gladbach, Germany).

### 4.2. Single-Cell RNA Sequencing

#### 4.2.1. Experiment Set-Up

In 5 mL medium per sample, 3 × 10^6^ REC-1 cells grew for 48 h. Ibrutinib was added to the 48 h sample after seeding and to the 6 h sample 6 h prior to harvest obtaining a final drug concentration of 400 nM. The Ctr sample stayed untreated. After harvest, the cells were washed with phosphate-buffered saline (PBS), stained with propidium iodide (2 µg/mL), and 30,000 cells were sorted by FACS (ARIA III, BD Biosciences, San Jose, CA, USA) to exclude possible cell debris, apoptotic cells, and doublets (Figure 1A). After counting the cells using trypan blue staining, they were loaded into the 10x Genomics Chromium (10x Genomics, Pleasanton, CA, USA). To generate the libraries, the Chromium Single Cell 3′ Kit v2 Chemistry was applied and all steps were performed following the recommended 10x Genomics protocol. For the quantification of the libraries a QubitTM 3.0 Fluometer (Thermo Fisher) was utilized and a 2100 Bioanalyzer with High Sensitivity DNA kit (Agilent Technologies, Waldbronn, Germany) was used for quality check. The reads were sequenced to a length of 100 bp in paired-end format on a S1 flowcell with a Novaseq 6000 (Ilumina, San Diego, CA, USA).

The two biological replicates were generated separately in an interval of three months from individually thawed and cultured REC-1 cells. Owing to the working/master stock system, the risk of cross-contamination of an originally purchased cell line is reduced.

#### 4.2.2. Data Analysis

The Cell Ranger software (version 3.0.1; 10x Genomics) was used to generate FASTQ files, to align reads (GRCh38 as a reference genome), to aggregate the data sets (default settings with depth normalization of data), and to create the feature-barcode matrices. The resulting raw data was further analyzed in R (version 3.6.0, https://www.r-project.org/, accessed on 15 January 2021) with the R package Seurat (version 3.0.2, manufacture, city, abbreviated state (for USA/Canada), country) adhering to the default settings unless otherwise specified [52].

To get rid of low-quality cells, thresholds for UMI (unique molecular identifier as a measure for mRNA content), gene, and mitochondrial gene content were adjusted (see Table S1). The data included for downstream analysis underwent log normalization. Highly variable features were identified serving as input for principal component analysis, a linear dimension reduction technique. The first 40 principal components were selected to cluster the cells (graph-based clustering). Different resolution parameters were tested to ascertain appropriate clustering. The uniform manifold approximation and projection (UMAP) served as a non-linear reduction technique to visualize the single-cell datasets. Differentially expressed genes (DEGs) were computed by the FindAllMarkers (log fold change > 0.1) function applying the Wilcoxon Rank Sum test (default). The heatmaps were created using the R packages ComplexHeatmap and Circlize [53,54].

#### 4.2.3. Batch Correction and Cell Cycle Regression

The aggregated dataset of the two replicates of Ctr were corrected for batch effects applying Canonical Correlation Analysis. Therefore, the standard integration workflow offered by Seurat was adapted to the analysis and performed after the quality control step [55].

Cell cycle regression reduced the effect of different cell cycle phases on the heterogeneity of the data. Each cell received a score based on canonical markers for S or G2/M phase and was then assigned to one of them. Cells that did not express any of these G2/M or S phase genes were categorized as G1 cells or rather resting cells. During data scaling, the impact of cell cycle genes was regressed out of the data according to Seurat’s cell-cycle scoring and regression approach [56].

#### 4.2.4. ClusterProfiler

To investigate upregulated Kyoto Encyclopedia of Genes and Genomes (KEGG) pathways or characteristic gene ontologies (biological processes), the R package ClusterProfiler (version 3.14.3) was applied [57]. The differentially expressed genes with a log fold change > 0.1 and adjusted *p*-value < 0.001 computed by Seurat’s FindAllMarkers function were considered for the analysis.

#### 4.2.5. SCENIC Analysis

Gene regulatory networks in the scRNA-seq data were analyzed by the R package SCENIC (version 1.1.2.2) [58]. The raw count matrix of the combined data set including both replicates of Ctr, 6 h, and 48 h was used as input after exclusion of low-quality cells (subpopulation E was not included due to low information content). Default settings were maintained for analysis. To identify possible regulons by RcisTarget, the hg19 motif ranking database for the human species offered by SCENIC was applied. Transcription factor modules with more than 10 mapped genes were kept for further analysis (suffix “_extended” indicated “low confidence” annotations). For exploration of gene regulatory networks, the regulon activity data from SCENIC (Figure 4A) was visualized based on the clustering shown in Figure 3A.

### 4.3. Cell Cycle Analysis

Cells were incubated as indicated, harvested, and resuspended in PBS. The cell suspension was added dropwise to ice-cold absolute ethanol to a final ethanol concentration of 70% while gently vortexing. The samples were stored in a freezer at −20 °C overnight. After centrifugation, the cells were washed with PBS and then incubated with propidium iodide (PI) solution (0.05 mg/mL propidium iodide in PBS; 0.1% Triton X; 0.2 mg/mL RNAse A (7000 units/mL)) at 37 °C for 40 min. The samples were diluted with PBS, centrifuged, and resuspended in PBS for analysis by flow cytometry (FACS Canto II, BD Biosciences) measuring PI signal in linear mode. The data was analyzed with Flowing Software (version 2.5.1, Perttu Terho, Turku Centre for Biotechnology, University of Turku, Turku, Finland, in collaboration with Turku Bioimaging).

### 4.4. Western Blot

Cells were harvested after incubation periods as indicated, washed with PBS, and lysed in lysis buffer (20 mM HEPES, pH 7.9; 350 mM NaCl; 1 mM MgCl_2_; 0.5 mM EDTA, pH 8.0; 0.1 mM EGTA, pH 8.0) supplemented with protease and phosphatase inhibitors for 20 min on ice. Protein concentrations were quantified by Bradford assay. 4× loading buffer (200 mM Tris, pH 6.8; 40% glycerol; 8% SDS; 4% β-mercaptoethanol; 50 mM EDTA, pH 8.0; 0.01% bromophenol blue) was added and the lysates were boiled to 96 °C for 5 min. After separation by dodecyl sulfate polyacrylamide gel electrophoresis, the proteins were blotted on nitrocellulose membranes and visualized by Ponceau S (Sigma-Aldrich, St. Louis, MO, USA) staining. Before incubation with primary and HRP-conjugated secondary antibodies, the membranes were blocked in Tris-buffered saline with 5% milk powder and 0.1% Tween 20. The protein bands were detected by Thermo Scientific SuperSignal West Pico Chemiluminescent Substrate (Thermo Fisher) and then visualized on Amersham Hyperfilm ECL (GE Healthcare, Freiburg, Germany). The area of protein bands determined by Image J software was used to perform normalization to β-actin and to calculate relative expression subsequently [59].

### 4.5. Extracellular Flux Analysis

The Seahorse XF 96 Metabolic Flux Analyzer (Seahorse Bioscience, Lexington, MA, USA) was used to measure the oxygen consumption rate (OCR) and extracellular acidification rate (ECAR). Cells entered the analysis after culturing as indicated. After harvesting, the cells were resuspended in prewarmed Seahorse medium (Agilent Seahorse XF RPMI Medium pH 7.4, supplemented with 10 mM Seahorse XF d-Glucose and Seahorse XF 2 mM l-Glutamine). Cells were counted by the Countess Automated Cell Counter (Thermo Fisher) and 1.1 × 10^5^ viable cells per well (6 technical replicates per condition) were plated into a poly-d-lysine (P6407, Sigma-Aldrich) coated Agilent Seahorse XF96 Cell Culture Microplate (Seahorse Bioscience). To immobilize the cells on the bottom, the plate was centrifuged at 500× *g* for 5 min. Subsequently, the plate was put in a non-CO_2_ incubator at 37 °C for 30 min, 130 µL of Seahorse medium were added to each well and the plates were returned for another 25 min to the non-CO2 incubator at 37 °C until analysis. OCR and ECAR were measured five times with 30 s intervals of mixing.

### 4.6. Flow Cytometric Analysis of Cell-Surface Antigens

After incubation, cells were washed, and resuspended in autoMACS Running Buffer (Miltenyi Biotech) before staining with antibodies and isotype control (dilution according to the manufacturer’s instructions) at 4 °C for 10 min. Then, the cells were washed and resuspended in autoMACS Running Buffer for flow cytometric analysis. The data was analyzed with Flowing Software using GeoMean as mean fluorescence intensity (MFI) to determine the shifts in surface antigens. The isotype control values of every condition were subtracted, e.g., MFI(CD52, DMSO) = GeoMean(CD52, DMSO) − GeoMean(isotype control, DMSO).

### 4.7. Statistical Analysis

Error bars represent the standard error of the mean of three independent experiments. After testing the homogeneity of variances by F-test, statistical significance was determined by the Student’s *t*-test either for equal or unequal variances using excel (Microsoft, Redmond, WA, USA); *p*-values ≤ 0.05 (* *p* ≤ 0.05, ** *p* ≤ 0.005) were considered statistically significant (ns = not significant).

## 5. Conclusions

Our single-cell sequencing approach allowed us to evaluate the changeover of the heterogeneous transcriptome profile of a mantle cell lymphoma cell line triggered by 48 h ibrutinib treatment. The surviving cells adjusted their gene expression to deal with the drug-induced pressure on crucial signaling pathways. We here described the upregulation of B cell receptor subunits, crosstalk with the microenvironment, upregulation of *CD52*, and metabolic reprogramming towards reliance on oxidative phosphorylation as potential mechanisms favoring ibrutinib resistance. Based on these findings and future continuative studies, novel therapeutic approaches may improve the outcome of MCL patients in ibrutinib-relapse settings.

## Data Availability

The scRNA-seq datasets are available in the Gene Expression Omnibus repository, GSE162234.

## Supplementary Materials

The following are available online at https://www.mdpi.com/1422-0067/22/5/2276/s1.
