# Supplementary material for: Time-Resolved scRNA-Seq Tracks the Adaptation of a Sensitive MCL Cell Line to Ibrutinib Treatment
Source: Int J Mol Sci. 2021 Feb 25;22(5):2276. doi: 10.3390/ijms22052276 (PMC7956352; doi:10.3390/ijms22052276)
Supplement: Supplementary file 1 [file ijms-22-02276-s001.zip › Supplementary Information.docx]

# Supplementary information

**Supplementary Tables**

Table S1: Thresholds for quality control.

Table S2: Marker genes for all clusters of the integrated and cell cycle regressed Ctr data set.

Table S3: Marker genes for the combined data set comparing cells of Ctr, 6 h, and 48 h.

**Figure S1**

Figure S1: Aggregation, cell cycle regression, and integration procedure.

Workflow for the aggregation, cell cycle regression, and integration process shown for Ctr (the used software tools are named in brackets).

**Figure S2**

Figure S2: Expression features of cluster 4 and a glycolysis/hypoxia related subgroup in the Ctr sample.

A) Expression plots (log normalized) of marker genes from Cluster 4 of Ctr. B) Expression plots (log normalized) of marker genes of the glycolysis/hypoxia related subgroup of Ctr cells. C) Uniform Manifold Approximation and Projection (UMAP) representation of cluster 4 (Ctr) and heatmap showing the top10 marker genes after re-clustering at resolution 0.6.

**Figure S3**

Figure S3: Adaptations in metabolism triggered by ibrutinib.

A) Separated analysis of the subpopulations A, B, and D of the combined analysis. The Uniform Manifold Approximation and Projection (UMAP) representations of treatment, clusters after re-clustering (A and B at resolution 0.2, D at resolution 0.3), and the Kyoto Encyclopedia of Genes and Genomes (KEGG) pathway analysis including genes with a Log fold change > 0.1 and adjusted *p*-value < 0.001. B) Log normalized expression of hypoxia associated genes across treatment: *BNIP3* and *DDIT4*. C) Violin plots of glycolysis associated genes: *IGFBP2* and *PGK1*.

**Figure S4**

Figure S4: Surface antigen expression across ibrutinib treatment.

A) Violin plots of surface antigen expression levels across ibrutinib treatment of the single-cell sequencing data. B) Flow cytometric data of the surface antigens *CD52*, *CD37*, and *CD40*. Mean fluorescence intensity (GeoMean) shown for sensitive REC-1 and resistant MAVER-1 after 3 d ibrutinib treatment (400 nM) and DMSO control (n = 3, * *p* ≤ 0.05, ** *p* ≤ 0.005, ns = not significant).

**Figure S5**

Figure S5: Entire heatmap of gene regulatory networks across ibrutinib treatment.

Gene regulatory networks in the eleven clusters of the combined analysis (clustering is shown in Figure 3A). Numbers in brackets indicate the amount of genes forming the gene regulatory networks**.**
