# Supplementary figures and images for: Time-Resolved scRNA-Seq Tracks the Adaptation of a Sensitive MCL Cell Line to Ibrutinib Treatment
Source: Int J Mol Sci. 2021 Feb 25;22(5):2276. doi: 10.3390/ijms22052276 (PMC7956352; doi:10.3390/ijms22052276)

Figure S1

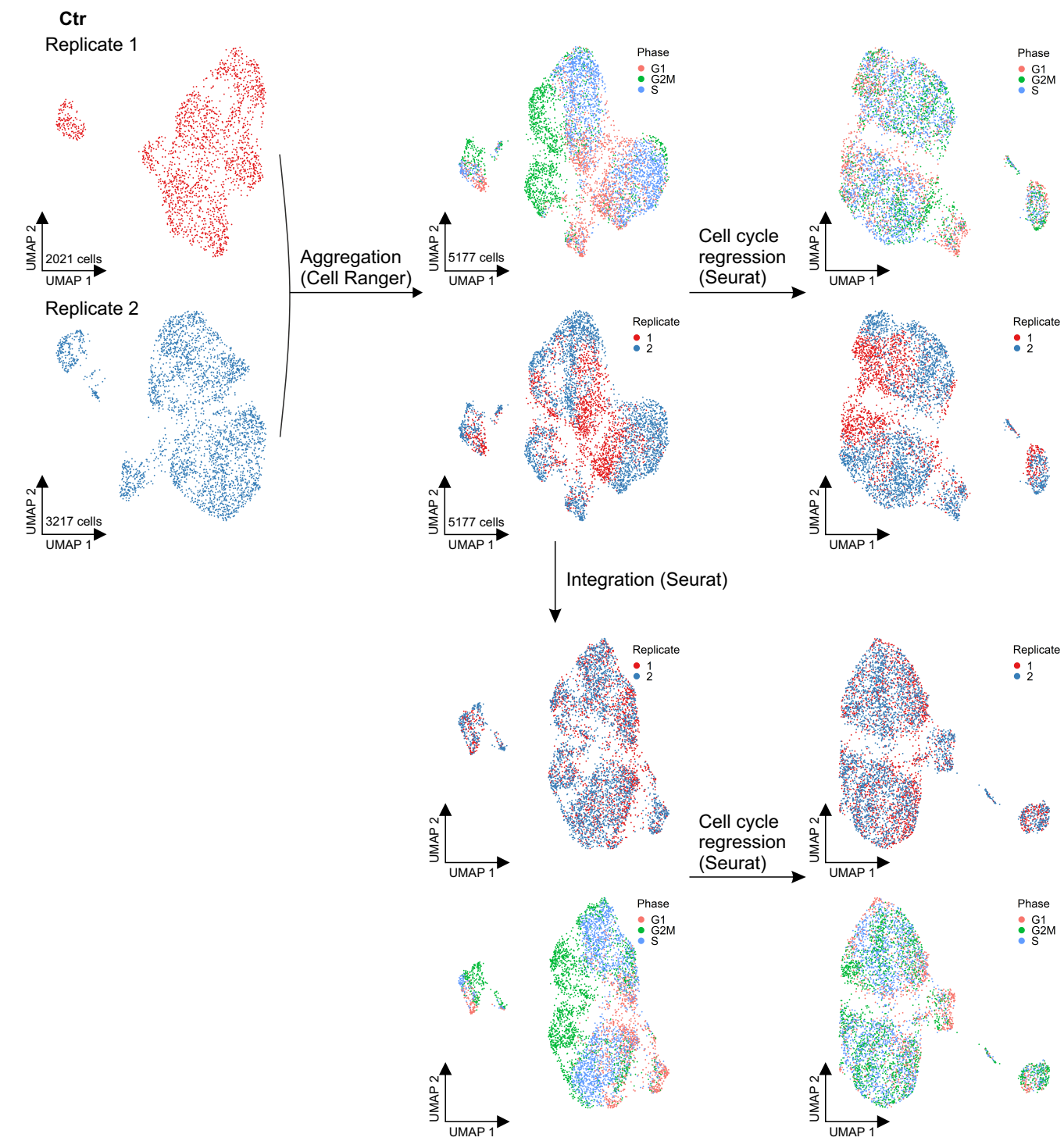

Supplement: Supplementary file 1 [file ijms-22-02276-s001.zip › Figure S1.pdf]

Figure S2

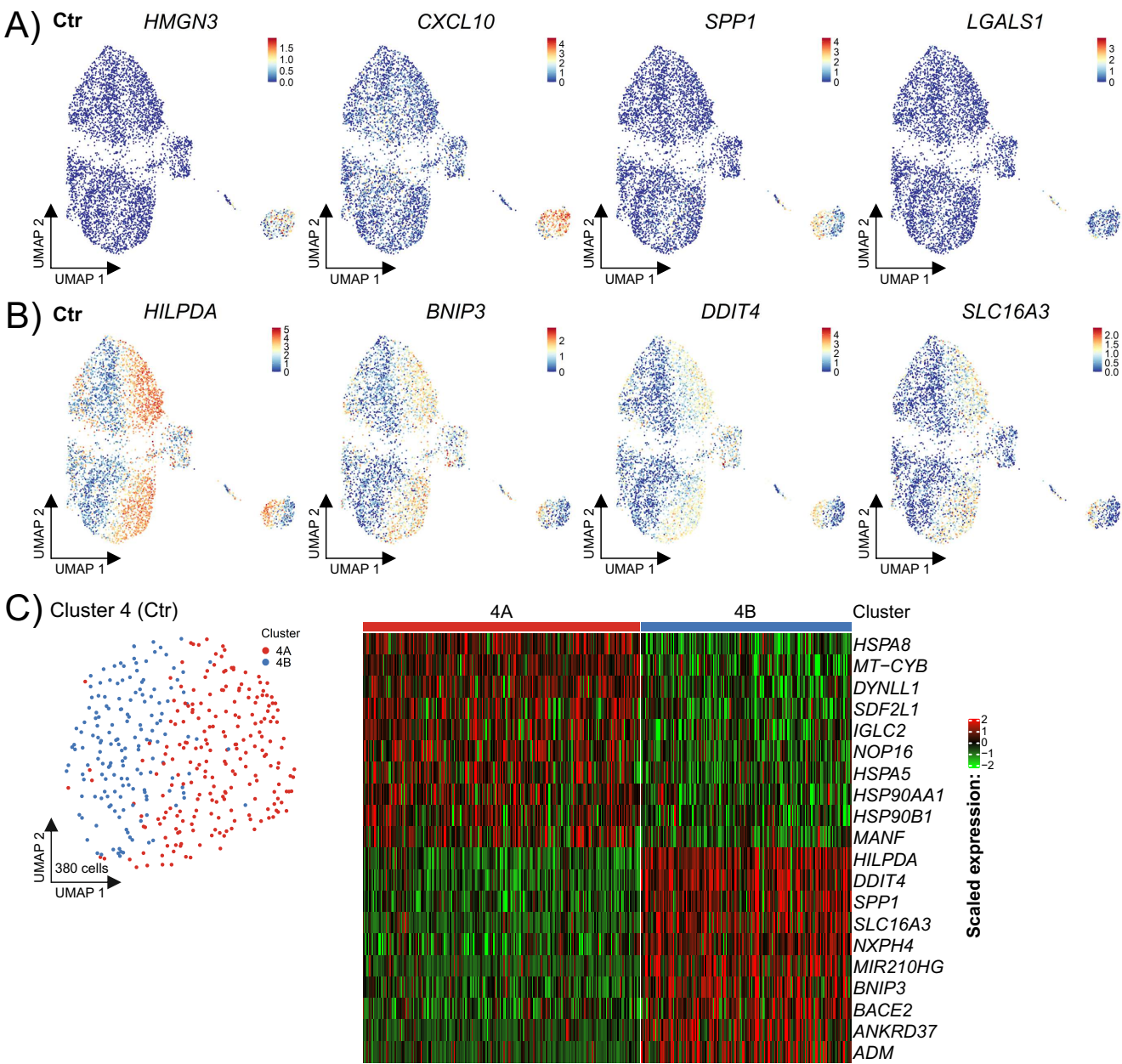

Supplement: Supplementary file 1 [file ijms-22-02276-s001.zip › Figure S2.pdf]

Figure S3

A) Subpopulation A

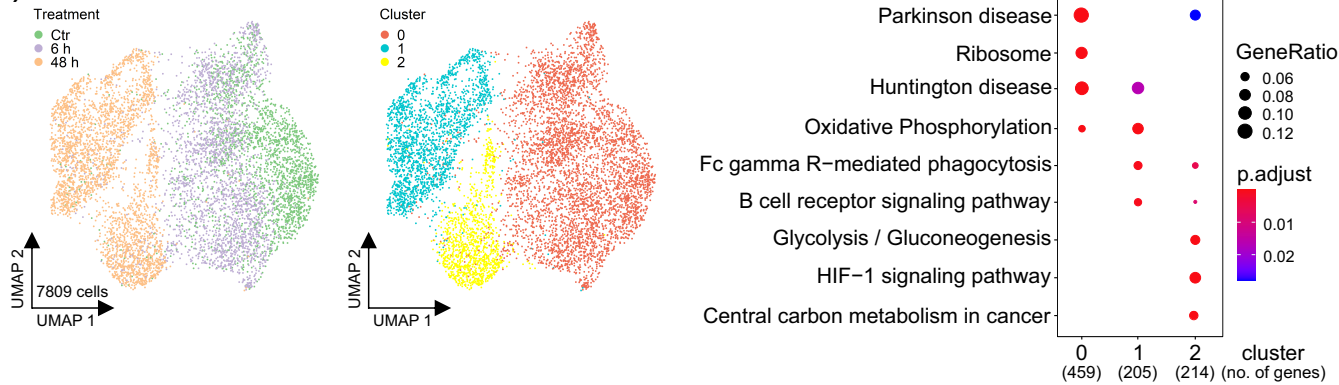

Subpopulation B

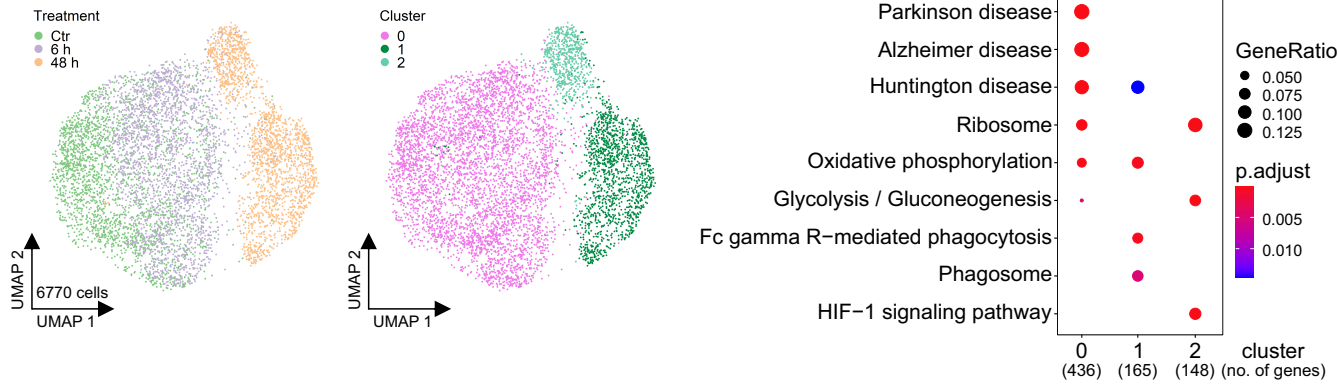

Subpopulation D

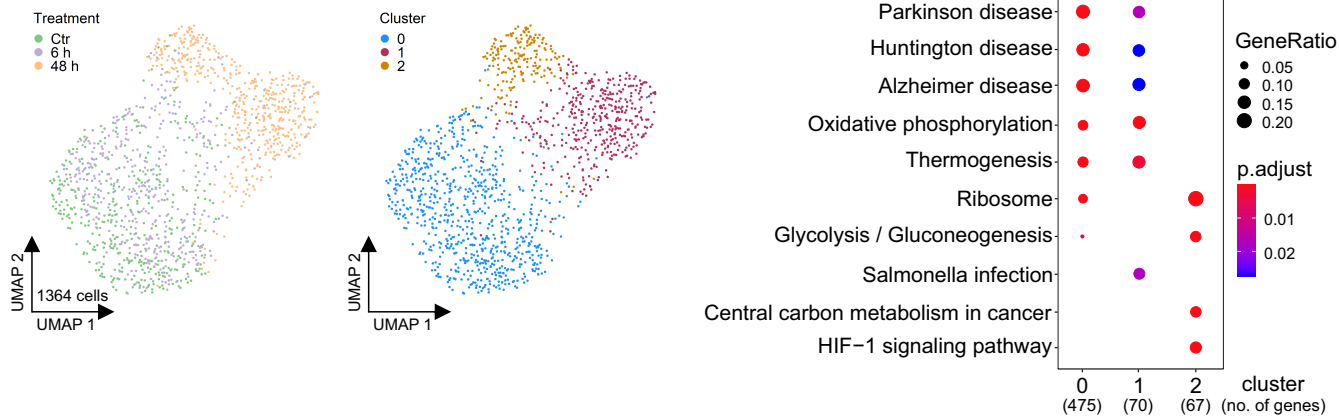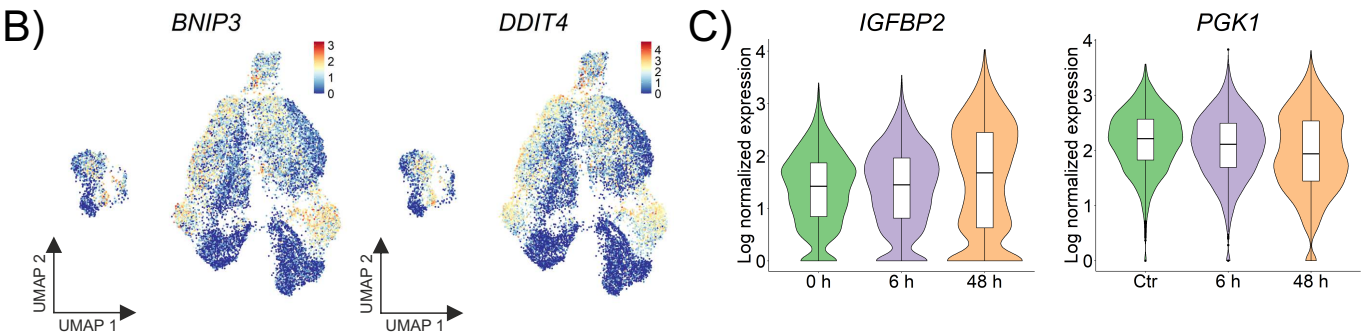

Supplement: Supplementary file 1 [file ijms-22-02276-s001.zip › Figure S3.pdf]

Figure S4

A)

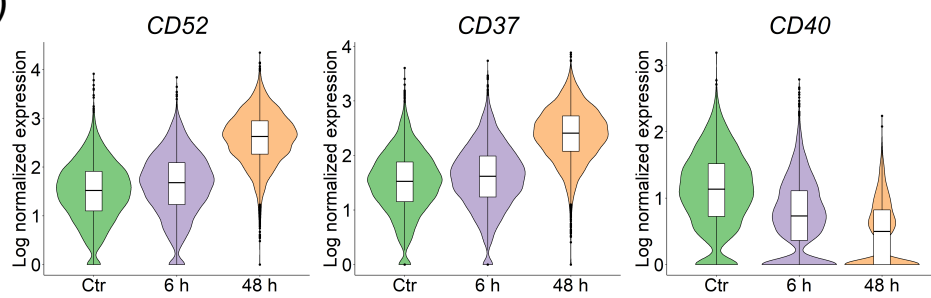

B)

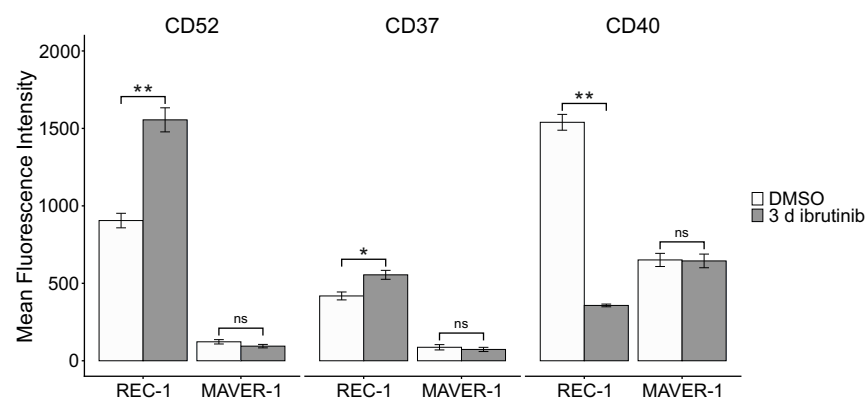

Supplement: Supplementary file 1 [file ijms-22-02276-s001.zip › Figure S4.pdf]

Figure S5

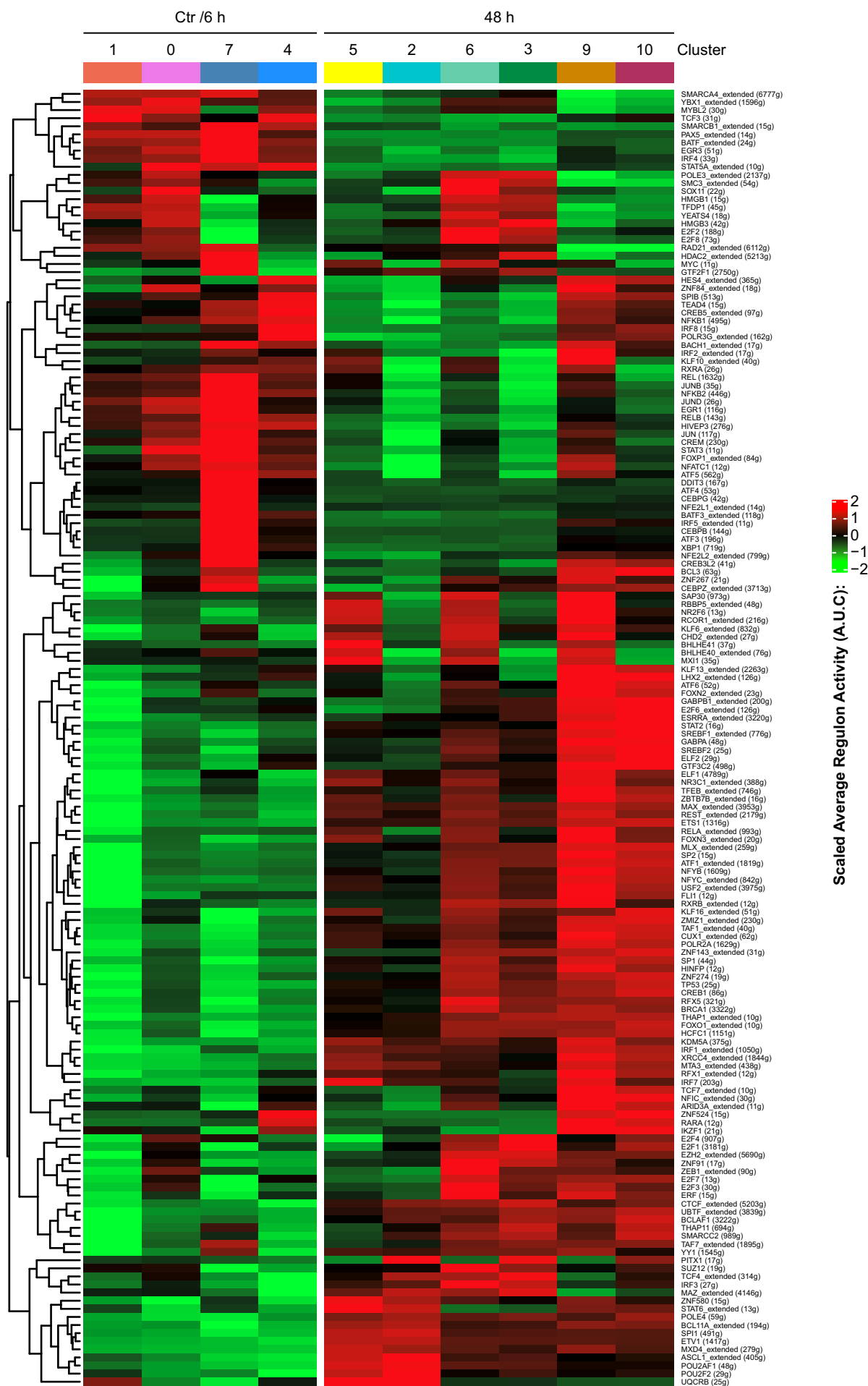

Supplement: Supplementary file 1 [file ijms-22-02276-s001.zip › Figure S5.pdf]
